# Exploring the Interaction of 3-Hydroxy-4-pyridinone Chelators with Liposome Membrane Models: Insights from DSC and EPR Analysis

**DOI:** 10.3390/molecules29245905

**Published:** 2024-12-14

**Authors:** Luísa M. P. F. Amaral, Tânia Moniz, Maria Rangel

**Affiliations:** 1 REQUIMTE, LAQV, Departamento de Química e Bioquímica, Faculdade de Ciências, Universidade do Porto, R. do Campo Alegre, 4169-007 Porto, Portugal; tmoniz@icbas.up.pt; 2 REQUIMTE, LAQV, Instituto de Ciências Biomédicas de Abel Salazar, Universidade do Porto, Rua Jorge Viterbo Ferreira, 228, 4050-313 Porto, Portugal; mrangel@icbas.up.pt

**Keywords:** 3-Hydroxy-4-pyridinone, differential scanning calo-hydroxy-4-pyridinone derivatives rimetry (DSC), electron paramagnetic resonance (EPR), liposome, membrane interaction

## Abstract

In this study, we synthesized a series of 3-hydroxy-4-pyridinone (3,4-HPO) chelators with varying lipophilicity by modifying the length of their alkyl chains. To investigate their interaction with lipid membranes, we employed differential scanning calorimetry (DSC) and electron paramagnetic resonance (EPR) spectroscopy using dimyristoylphosphatidylcholine (DMPC) and palmitoyloleoylphosphatidylcholine (POPC) liposomes as membrane model systems. DSC experiments on DMPC liposomes revealed that hexyl-substituted chelators significantly altered the thermotropic phase behavior of the lipid bilayer, indicating their potential as membrane property modulators. EPR studies on DMPC and POPC liposomes provided detailed insights into the depth-dependent effects of chelators on membrane fluidity. Our findings highlight the crucial role of alkyl chain length in determining the interaction of 3,4-HPO chelators with lipid membranes and offer valuable insights for the design of lipid-interacting therapeutic agents based on this scaffold.

## 1. Introduction

Understanding drug-membrane interactions is crucial for addressing the complexities of many diseases, such as cancer [1,2], neurodegenerative disorders [3,4], infectious diseases [5,6], and metabolic diseases, particularly diabetes [7,8,9]. Drugs that target membrane structures or transporters, such as liposomal formulations or membrane-penetrating peptides, have shown promising effects on overcoming biological barriers, delivering drugs to specific tissues, and enhancing drug stability [10]. On the other hand, lipophilicity is one of the factors that may influence biological activity and is therefore an important parameter to determine for newly obtained compounds as drug candidates. Drugs that poorly interact with cell membranes may exhibit low permeability, reducing their ability to reach intracellular targets. Conversely, drugs that strongly interact with membranes may accumulate excessively, potentially leading to toxicity. Thus, fine-tuning drug-membrane interactions can optimize drug delivery, enhance therapeutic outcomes, and minimize side effects.

The 3-hydroxy-4-pyridinone (3,4-HPO) chelators are widely recognized for their significant pharmaceutical and environmental applications [11,12,13,14]. Their strong chelating abilities towards relevant transition metal ions underscore their potential use. Their applications include PET imaging [11], detection and quantification of environmental and biologically important species such as metal ions [12,13], and treatment of diseases associated with metal ion overload [14].

The 3,4-HPO chelators offer a solution by binding these ions with high stability, thereby mitigating their toxicity and aiding in their regulation within the body. One of the key advantages of 3,4-HPO chelators is their tunable hydrophilic/lipophilic balance. By modifying substituents on the heterocyclic ring of these chelators, their solubility properties can be fine-tuned to optimize their interaction with biological membranes. This adjustment is crucial for enhancing the bioavailability and efficacy of these chelators in therapeutic applications, ensuring they can effectively reach and interact with their targets. Chelators from this family play a crucial role in medicine, particularly in the treatment of metal overload diseases such as β-thalassemia and Wilson’s disease, where excess iron and copper, respectively, need to be effectively managed [15]. These metal ions are essential for various biological processes but can be harmful in excess.

Also, the 3,4-HPO ligands have been reported as relevant platforms for the design of other metal complexes, such as zinc(II) and vanadium(IV/V) chelates, which have been studied as potential anti-diabetic drugs [16,17]. In this particular case, the biological activity of these complexes may be due to their interaction with phosphatase and kinase enzymes involved in the insulin signaling cascade, which are crucial for the lowering of blood glucose levels [18]. Since these key players enzymes are located in the cytoplasm, the interaction and permeation across the membrane of the cells are required steps. Thus, it seems relevant to study the ability of these molecules to interact with cellular membranes. Particularly for the metal ion complexes of 3,4-HPO, there is a relationship between the lipophilicity of the ligands and that of the corresponding metal ion complexes, suggesting identical solvation effects for the ligands and complexes [19]. For this reason, it seems plausible to first consider the study of the drug-membrane interaction by using the ligands, as a preliminary approach, which can be taken as a prediction of the interaction of their respective metal chelates with the model membranes, as already seen in previous studies [20,21,22]. Moreover, this type of study will be useful for the further selection of the most active ligands to be used in the preparation of novel metal ion chelates with potential biological activity.

Our group already performed investigations on the effect of vanadium 3,4-HPO complexes as anti-diabetic drugs, and the results pointed out that two pyridinone complexes, VO(mpp)_2_ [bis(3-hydroxy-2-methyl-4(1*H*)-pyridinone)VO] and VO(empp)_2_ [bis(2-ethyl-3-hydroxy-1-methyl-4(1*H*)-pyridinone)VO], have a significant in vitro insulin-mimetic activity [17]. The in vivo studies considering zinc 3,4-HPO complexes pointed out the relevance of the lipophilicity of the ligands/chelates for their biological activity and pointed out the higher effect of the compound Zn(dmpp)_2_ in the lowering of glucose for the treatment of type I diabetes [16].

**Figure 1:**
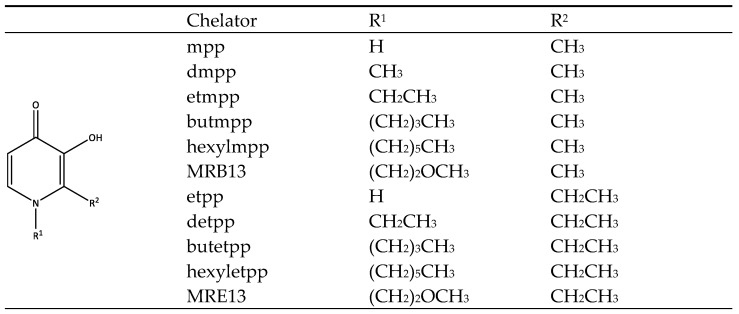
Formulae and abbreviations of the 3-hydroxy-4-pyridinone chelators studied in this work.

In the present study, we studied the interaction of a set of 3,4-HPO ligands, including some recently developed ones, considering two different pyrone precursors: maltol (R^1^ = CH_3_) and ethylmaltol (R^1^ = CH_2_CH_3_) (Figure 1). This is the first step of a full investigation regarding this family of molecules, and based on the results herein, a more rational design can be performed, which aims to synthetize novel zinc and vanadium 3,4-HPO complexes to be further inspected regarding their potential anti-diabetic activity. The formulae of compounds are shown in Figure 1. Different types of liposomes may be employed in EPR and DSC studies due to their distinct analysis requirements. In EPR, probes like 5-doxyl-stearic acid (5-DSA) and 16-doxyl-stearic acid (16-DSA) are introduced within the liposomes to interact with the lipid bilayer. EPR provides indirect data by analyzing the perturbations in the signal from spin-label probes embedded in the lipid bilayer. This approach reveals information about lipid dynamics and fluidity at specific points within the membrane, offering a localized view of the bilayer environment. In contrast, DSC captures data directly, as it measures the overall heat flow required to induce phase transitions in the lipid bilayer. This offers a macroscopic understanding of the thermodynamic properties of the membrane, such as phase transition temperature (*T*_m_), enthalpy changes, and cooperativity. These complementary methods allow for a detailed understanding of the lipid bilayer’s response to various chelators, with DSC giving a broad view of thermodynamic stability and EPR providing insights into localized fluidity changes induced by the interaction of the chelators with specific membrane regions. The DSC study was conducted with DMPC liposomes, which exhibit a phase transition at approximately 24.5 °C, allowing for accurate measurement of the gel-to-liquid crystalline phase transition temperature using this technique. For the EPR study, DMPC and POPC liposomes were used.

## 2. Results and Discussion

### 2.1. Liposome Size Determination by DLS

The size distribution of the liposomes, (mean diameter and polydispersity index (PDI) was determined using dynamic light scattering (DLS). To assess the impact of chelator interactions on liposome size, samples containing the chelators that penetrate in the membrane were also analyzed. The addition of the compounds butmpp, hexylmpp, butetpp and hexyletpp, did not significantly change the particle size and PDI, as shown in Table 1.

### 2.2. DSC Study of Chelator/DMPC Liposome Mixtures

**Figure 2:**
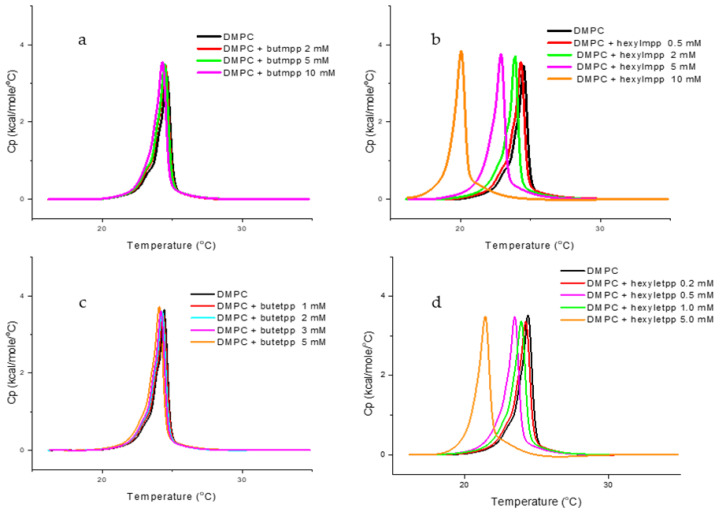
The DSC heating thermograms for the main phase transition of DMPC unilamellar vesicles (LUVs), in the presence and absence of different concentrations of chelators: (**a**) butmpp; (**b**) hexylmpp; (**c**) butetpp; and (**d**) hexyletpp.

To investigate the interaction of the chelators with lipid membranes, we used dimyristoylphosphatidylcholine (DMPC) large unilamellar vesicles (LUVs) as a model system due to its high abundance in mammalian membranes and relatively easy preparation protocol [23]. Chelators stock solutions were prepared in buffer solution (10 mM HEPES, 150 mM NaCl, pH 7.4). The chelator solution was added to the DMPC suspension to achieve a final concentration of 5 mM, and the mixture was incubated for 1 h at 37 °C to allow equilibration. These initial experiments were conducted to obtain insight into the type of interaction of the synthesized chelators with the DMPC liposomes. By testing the chelators at a high concentration of 5 mM, we aimed to identify those that caused detectable changes in the thermotropic properties of the liposome membranes, as observed in the DSC thermograms (Figure 2). Table 2 presents the thermodynamic parameters determined based on the heating scans: transition temperature (*T*_m_), enthalpy change (Δ*H*), and the corresponding half-width at half-height (Δ*T*_1/2_) for DMPC in the presence of the chelator in the described conditions.

Literature data indicate that pure DMPC liposomes exhibit a prominent and sharp main endothermic transition around 24 °C, which corresponds to the temperature at which DMPC undergoes a phase transition from the gel (Lβ′) phase to the liquid-crystalline (Lα) phase, also known as the melting temperature, *T*_m_ [24,25,26]. Δ*T*_1/2_ indicates how wide the peak is at half of its maximum height and is a measure of the cooperativity of the phase transition. For pure lipids of DMPC, the pre-transition usually either cannot be observed for the LUVs, or it appears as a “shoulder” superimposed on the main phase transition curve.

The DSC data (Table 2) show that increasing the alkyl chain length, starting from butyl-derivatives, decreases the melting temperature (*T*_m_) of DMPC liposomes. This suggests that longer alkyl chains destabilize the membrane structure, reducing the temperature required for the gel-to-liquid-crystalline phase transition.

Interestingly, the enthalpy and cooperativity parameters remained mostly unchanged, indicating that the overall energy of the phase transition and the sharpness of this transition were not significantly affected. Only a slight increase in enthalpy was observed for hexylmpp. However, although the Δ*H* values reported here provide a general trend, they should be interpreted with caution. It is not possible to definitively conclude whether subtle changes in Δ*H* have occurred, as the experimental method may not have the necessary precision to detect them.

The constant enthalpy suggests that the bilayer’s structural integrity is maintained, but the organization of the lipid tails is influenced by hydrophobic interactions between the alkyl chains and the lipid tails. The progressive decrease in *T*m with longer chains could be linked to an increase in the disorder of the lipid tails, as the larger hydrophobic moieties disturb the packing of DMPC, weakening the van der Waals interactions between lipid molecules. In fact, the compounds can interact with lipids in biomembranes intercalating between the flexible acyl chains of lipids causing *T*m variations but no Δ*H* changes [27].

For DMPC/hexylmpp and DMPC/hexyletpp mixtures, a marked reduction in *T*_m_ was observed compared to the other derivatives. The main peak moves from *T*_m_ = 24.4 °C of pure DMPC to 22.5 °C and 21.5 °C, respectively, suggesting that the longer hexyl chain has a pronounced effect on the lipid bilayer structure.

The overall data also reveals that the changes in the thermodynamic parameters were solely influenced by the different R^1^ substituents on the pyridine ring, while the R^2^ substituents had little to no effect on the studied parameters. Therefore, it can be concluded that both groups of ligands tested, derived from the two pyrones (with R^2^ = CH_3_ or CH_2_CH_3_, respectively), exhibit similar interactions with DMPC liposomes, with these interactions being dependent on the substituents on the nitrogen atom of the heterocyclic ring.

Subsequently, the chelators that influenced the thermotropic behavior of DMPC suspensions were further investigated to assess their effects at variable concentrations (0.5 to 10 mM). This allowed us to better understand the concentration-dependent impact of each chelator on the lipid bilayer, providing insights into the strength and nature of the chelator-membrane interaction. Figure 2 shows the DSC heating plots for selected DMPC/chelator mixtures. The thermodynamic parameters are depicted in Table 3.

Hexyl derivatives induced a gradual decrease in the main transition temperature (*T*_m_) with increasing concentration, while cooperativity and enthalpy remained almost unchanged, for the tested concentrations, which is consistent with the findings observed at 5 mM. This suggests that the hexyl group increases membrane fluidity without significantly altering the overall organization or energy of the phase transition, despite the lowered *T*_m_.

In contrast, butyl derivatives exhibited a much smaller effect on *T*_m_, which may suggest that the shorter alkyl chain has a less pronounced impact on the lipid bilayer’s phase transition. The minimal effect of the butyl group might be related to its lower hydrophobicity or less significant disturbance of the lipid packing compared to the longer hexyl chain.

Chelators like MRB13 and MRE13 had no effect on thermodynamic parameters, possibly due to their higher hydrophilicity given by the presence of polar atoms, particularly oxygen [13], which limits interaction with the hydrophobic liposome bilayer.

### 2.3. EPR Study of Chelator/POPC and Chelator/DMPC Liposome Mixtures

For the EPR studies, POPC and DMPC were used. Similarly to DMPC, POPC is extensively used as a mimic for eukaryotic membranes and, thus, it can represent the human cell membrane, being, in fact, the most common phospho-lipid type [28]. POPC is a more fluid bilayer at room temperature (*T*_m_ = −2 °C) due to the unsaturation in its acyl chain. DMPC has a well-defined phase transition temperature (around 24 °C), being in a gel-solid state below it and fluid above. Selecting 37 °C for DMPC ensures it is in the fluid phase, simulating physiological conditions and enabling comparisons between the models.

**Figure 3:**
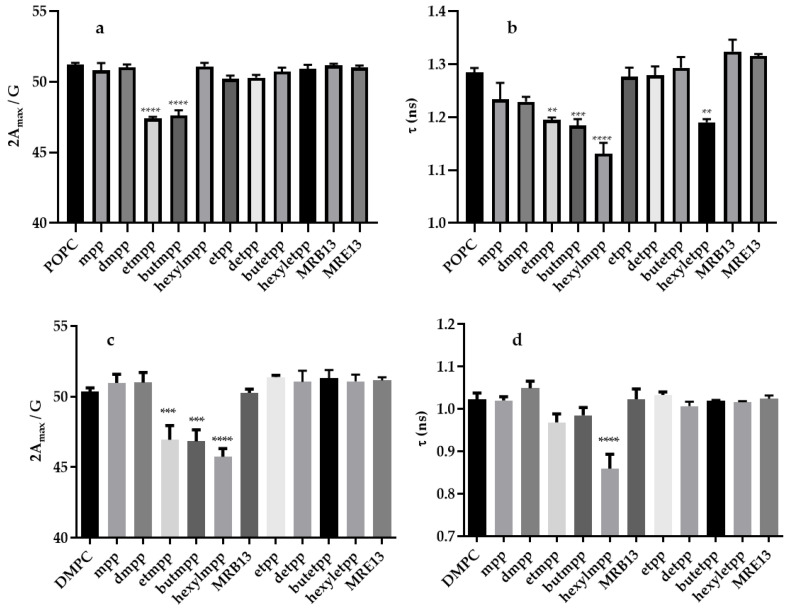
Hyperfine splitting (2A_max_) (**a**,**c**) rotational correlation time (τ) (**b**,**d**) obtained for POPC (**a**,**b**) and DMPC (**c**,**d**) liposomes in the absence (control) or in the presence of the chelators. The 5-DSA (**a**,**c**) and 16-DSA (**b**,**d**) spin probes have been used to study interaction of the compounds in the hydrophilic and hydrophobic region of the lipossomes, respectively. The data represent the mean ± standard error (SE) from three replicates. Statistical analysis was performed using one-way ANOVA. ** *p* ≤ 0.01; *** *p* ≤ 0.001; **** *p* < 0.0001.

EPR studies with POPC and DMPC liposomes labeled with 5-DSA and 16-DSA spin probes provide additional insights into how the chelators modulate membrane fluidity (see Figure 3) and allow the determination of the site of the interaction within the membrane. The results indicate that etmpp and butmpp alter the membrane fluidity of both liposomes, primarily affecting the region near the polar head groups (as detected by the 5-DSA probe). On the other hand, hexylmpp only influences the head group region of DMPC (Figure 3a,c).

Interestingly, the 16-DSA probe, located deeper within the lipid bilayer, exhibits distinct behavior in the two liposome types. While different compounds (etmpp, butmpp, hexylmpp, and hexyletpp) induce changes in POPC, only hexylmpp significantly impacts DMPC (Figure 3b,d). The selective modifications detected by the 5-DSA and 16-DSA probes highlight the role of both the liposome composition and the structural properties of the compounds. In DMPC and POPC liposomes, the effects detected by the 5-DSA probe suggest that etmpp and butmpp interact predominantly near the polar head group region, which is consistent with their moderate hydrophobicity and likely localization closer to the bilayer interface. The unique response of the 5-DSA probe to hexylmpp in DMPC implies that this compound may insert into the bilayer differently, possibly due to its longer alkyl chain, which enhances its partitioning into the lipid bilayer while maintaining interaction with the polar head group region in this liposome.

The 16-DSA probe results, which monitor deeper regions of the bilayer, show that in DMPC only hexylmpp induces detectable changes, suggesting that this compound has sufficient hydrophobic character to penetrate deeper into the bilayer core. This observation aligns with the tight packing and lower fluidity of DMPC bilayers, which may restrict the deeper insertion of shorter-chain compounds like etmpp and butmpp.

In contrast, the effects observed in POPC liposomes differ significantly. The 16-DSA probe shows changes in fluidity with etmpp, butmpp, hexylmpp, and hexyletpp, indicating that these compounds can penetrate deeper into the bilayer in this more fluid and unsaturated membrane system. This enhanced accessibility to the hydrophobic core in POPC compared to DMPC may result from the intrinsic differences in lipid packing and fluidity due to the unsaturated acyl chains in POPC. The fact that hexylmpp increases fluidity only in the 16-DSA region of POPC further supports its integration into the hydrophobic core without strongly affecting the more surface-exposed lipid regions.

Interestingly, the 2-ethyl derivatives, (Figure 1) show no interaction, a result that is not fully understood. This effect might result from weak or transient interactions between the derivatives and the membrane, enough to alter phase transition properties but insufficient to induce notable fluidity changes at the molecular level or could promote interactions at the membrane-water interface, influencing the overall packing and phase behavior without substantially affecting the bilayer’s dynamic properties probed by EPR. In fact, the presence of the ethylene group on the R^2^ position of the pyridinone ring, usually has an impact in the hydrophilicity of the ligands, as well as their respective metal ion complexes. We suppose that these ligands are probably less soluble in an aqueous medium, thus forming microscopic precipitates and, therefore, compromising the interaction of these molecules with liposome membranes. For example, a study considering iron(III) complexes derived from maltol (Fe(mpp)_3_ and Fe(dmpp)_3_) or from ethylmaltol precursors (Fe(etpp)_3_), showed that the methyl derivatives, (mpp and dmpp), have a superior effect on the fertilization of soybean plants, while the ethyl derivative (etpp) demonstrated a less pronounced effect [29]. Once again, biophysical studies pointed out that these differences were due to the lower hydrophilicity of the etpp-derived complex in comparison with the maltol-derived ones.

## 3. Materials and Methods

### 3.1. Chemicals

The 1,2-Dimyristoyl-sn-glycero-3-phosphocholine, (DMPC) and 1-hexadecanoyl-2-(9Z-octadecenoyl)-sn-glycero-3-phosphocholine, (POPC) were purchased from Avanti Polar Lipids (Alabama, AL, USA). The purity of lipids was over 99%, and for that reason, they were used without any further purification. The 1,2-dimethyl-3-hydroxy-4(1*H*)-pyridinone (dmpp), maltol, ethylmaltol, 5-DOXYL stearic acid ammonium salt (5-DSA), 16-DOXYL stearic acid (16-DSA), HEPES buffer (4-(2-hydroxyethyl)-1-piperazineethanesulfonic acid), and NaCl were all purchased from MERCK (Darmstadt, Germany). Reagents and solvents were purchased as reagent-grade and used without further purification unless otherwise stated.

### 3.2. Synthesis of the Chelators

The chelators used in this study were synthesized in our laboratory following previously reported methods [12,16]. The ligand MRE13 was first synthesized in the present study and its characterization is described in the Supplementary Materials (Figures S1–S3). Briefly, the 3-hydroxypyridin-4-one derivatives were prepared from the corresponding 3-hydroxypyran-4-ones in a three steps reaction: the first step is the protection of the hydroxyl group of 3-hydroxypyran-4-one; the second step is the reaction of the benzylated 3-hydroxypyran-4-one with a primary amine R1-NH_2_ to give the benzylated pyridinone; the third step is the catalytic hydrogenation to remove the protecting group, yielding the corresponding bidentate chelators as hydrochloride salts.

### 3.3. Preparation of Liposomes

Multilamellar liposomes (MLVs) were prepared by the hydration method. Briefly, the desired amount of DMPC or POPC powder was dissolved in a mixture of chloroform/methanol (87.4:12.6% (*v*/*v*)) to further obtain a suspension of 6 mM. The solution was placed in a round-bottomed flask and excess chloroform/methanol was evaporated using a gentle stream of nitrogen to form a lipid film. The residual solvent was eliminated by placing the film under a vacuum for at least 6 h. The lipid film was hydrated using a HEPES buffer solution (10 mM HEPES, 150 mM NaCl, pH 7.4), and prepared with Milli Q gradient Ultra-pure water (Millipore, Billerica, MA, USA). The hydration was performed while maintaining the solution in a water bath at approximately 10 °C above the lipid’s main phase transition temperature.

The MLVs formed were subjected to six freeze–thaw cycles, freezing in liquid nitrogen and thawing above the transition temperature, *T*_m_. LUVs were subsequently prepared from the MLVs using the extrusion method under an inert nitrogen atmosphere. The extrusion was carried out using two stacked polycarbonate filters with a pore size of 100 nm (Whatman, Nuclepore, Nova Jersey, NJ, USA) in a 10 mL stainless steel extruder (Lipex Biomembranes Inc., Vancouver, Canada) at a temperature approximately 10 °C above the gel-fluid transition temperature of the lipid system, with 15–20 passes through the filter. The lipid content in the liposomal suspension was determined by a spectrophotometric method as previously described [30]. For the EPR experiments, 1% (mol/mol) of each EPR spin probe (5-DSA or 16-DSA) (by addition of the desired volume of the probe solution in methanol) was incorporated into the lipid solution, and the same protocol previously described was followed.

The final liposome concentration used in the DSC and EPR experiments was 3 mM.

### 3.4. Vesicle Size Distribution

Measurements of the size and polydispersity of liposomes were obtained by using dynamic light scattering (DLS) with a Nano Zetasizer from Malvern Instruments (Malvern, UK). Five measurements were conducted above the transition temperature (*T*_m_) (37 °C) at a lipid concentration of 0.1 mM, utilizing a He-Ne laser (633 nm wavelength) as the light source and operating at a scattering angle of 173°. The resulting samples were monodispersed, exhibiting a particle size of approximately 110–120 nm and a polydispersity index (PDI) consistently below 0.1.

### 3.5. Differential Scanning Calorimetry (DSC)

DSC measurements were performed via a MicroCal VP-DSC microcalorimeter from Malvern (Worcestershire, UK), following the protocol established in the literature [31]. Briefly, prior to sample analysis, blank experiments were performed using a buffer in both cells to account for any baseline drift. A heating and cooling rate of 60 °C/h, in the 15–35 °C range for DMPC was used and each sample underwent three consecutive heating scans with the buffer in the reference cell to ensure reproducibility. Thermal cycles were conducted on three different samples to verify the consistency and reproducibility of the data. Transition temperatures (*T*_m_), enthalpy changes (Δ*H*), and the half-width at half-maximum (Δ*T*_1/2_) were determined by integrating the heat capacity versus temperature curve (Cp vs. T) after blank correction, using a linear baseline and the Microcal OriginTM software 7.0.

### 3.6. Electron Paramagnetic Resonance (EPR)

The EPR measurements were performed on a Bruker ELEXSYS E 500 spectrometer (Bruker, Billerica, Massachusetts, MA, USA), equipped with an ER4222SHQ resonator, available at Laboratório de Análise Estrutural, Centro de Materiais da Universidade do Porto (CEMUP; Porto, Portugal). The spectrometer was equipped with a Bruker N_2_ temperature controller (Bruker, Billerica, Massachusetts, MA, USA), ensuring a temperature stability of ±0.1 K throughout the experiments. The spectra were recorded using a microwave power of 25.18 mW, within a magnetic field range of 3285 G to 3435 G (150 G window), with a modulation amplitude of 4 G. The data were acquired over 1024 points, with a conversion time of 100 ms, and 6 scans. Three independent replicates were performed to ensure the reliability and reproducibility of the EPR studies.

The studies were conducted using unilamellar liposomes composed of POPC, at room temperature (21 °C), and DMPC, at 37 °C, to investigate the effects of the chelators on membrane fluidity. Two spin probes, 5-DSA and 16-DSA, were used to provide insight into different regions of the lipid bilayer. The 5-DSA probe, located near the polar head group region of the membrane, allowed for the assessment of alterations in the outer, more hydrophilic environment. In contrast, the 16-DSA probe, embedded deeper within the lipid bilayer, provided information on the hydrophobic core region of the membrane. By analyzing the changes in the EPR spectra of these probes, it was possible to determine how the tested compounds modulated the fluidity in both the polar and hydrophobic regions of the two bilayers, offering a detailed understanding of their impact on membrane dynamics. The determination of 2A_max_ and correlation time (τ) was calculated from the specific values directly determined in the spectra as previously described [29].

Statistical analysis of the results was performed using GraphPad Prism (version 8.0.1 for Windows; GraphPad Software Inc., San Diego, CA, USA). The obtained data from the three independent experiments were expressed as mean ± standard deviation (SD), and a one-way analysis of variance (ANOVA) with multiple comparisons was performed. Statistical significance was considered for *p* < 0.05.

## 4. Conclusions

The integration of the DSC and EPR results provides a comprehensive understanding of how the synthesized compounds interact with DMPC liposomes. The DSC data reveal that hexyl derivatives cause a gradual decrease in the main transition temperature (*T*m) of DMPC as their concentration increases, without significantly altering the enthalpy or cooperativity of the phase transition. This suggests that hexyl derivatives partially disrupt lipid packing, facilitating the gel-to-fluid phase transition while preserving the overall structural integrity of the bilayer. Butyl derivatives have only a minor effect on *T*m, indicating a less pronounced impact on the thermotropic properties of the lipid bilayer, consistent with their more surface-localized interactions. Compounds with shorter alkyl chains do not alter the thermotropic properties of DMPC, confirming their minimal interaction with the bilayer. Similarly, MRB13 and MRE13 show no effect on the DMPC thermotropic profile.

The EPR results with DMPC indicate that hexylmpp induces changes in membrane fluidity detectable by both the 5-DSA and 16-DSA probes, demonstrating its interaction with both the polar head group region and the hydrophobic core of the bilayer. In contrast, etmpp and butmpp affect membrane fluidity only near the polar head group region, as detected by the 5-DSA probe. The other compounds studied exhibit no interaction with the bilayer. These findings align with our previous studies on 3,4-HPO ligands and their Zn(II) complexes. Previously reported molecular dynamics simulations on DMPC bilayers demonstrated that ligand hydrophobicity significantly influences membrane partitioning and complex behavior. The most hydrophobic ligand, hexylmpp, exhibited the highest lipid partitioning, corroborating the herein-obtained experimental observations [32].

The discrepancy between the DSC and EPR results for the 2-ethyl pyridinone derivatives is an interesting observation. While the DSC data suggest a decrease in the melting temperature of DMPC, for butetpp and hexyletpp, EPR data indicate no significant change in membrane fluidity. A reduction in *T*m typically indicates a disturbance in the packing of the lipid bilayer, suggesting that the 2-ethyl derivatives interact with the DMPC membrane to some extent, destabilizing its ordered gel phase. The compounds might primarily interact with the DMPC membrane in the gel phase, leading to the observed reduction in *T*m measured by DSC. Once the membrane transitions into the fluid phase, as in the EPR experiments conducted at 37 °C, the interactions could weaken or become negligible, resulting in no detectable changes in membrane fluidity.

The EPR results with 5-DSA and 16-DSA probes showed that the compounds etmpp, butmpp, hexylmpp, and hexyletpp differentially affect membrane fluidity in POPC and DMPC liposomes, depending on the probe and lipid composition.

Results with the 5-DSA probe show the effect of etmpp and butmpp on membrane fluidity in POPC, while etmpp, butmpp, and hexylmpp showed this effect in DMPC. The enhanced effect in DMPC with longer alkyl chains suggests that these compounds interact more effectively with the lipid headgroup region in this more rigid membrane.

For the 16-DSA probe, etmpp, butmpp, hexylmpp, and hexyletpp show increased fluidity in POPC. The higher fluidity of POPC membranes allows these compounds to penetrate deeper into the liposome core, disturbing the hydrophobic packing. In DMPC, only hexylmpp increased core fluidity, likely because its longer alkyl chain may probably overcome the tighter packing of the saturated bilayer.

These results emphasize the role of alkyl chain length in altering the biophysical properties of membranes. Such modulations depend on lipid composition and could have implications for the design of chelators and their respective metal ion complexes to achieve specific biological activities. Chelators from this family or their respective complexes can modulate membrane permeability and influence membrane-associated processes, offering potential in targeted therapeutic applications.

## Figures and Tables

**Table 1:** Diameter (*d*) and polydispersity index (PDI) of the liposomes.

Chelator	d (nm)	PDI	d (nm)	PDI
	DMPC		POPC	
No chelator	112.2 ± 1.8	0.031 ± 0.009	115.0 ± 1.2	0.065 ± 0.020
butmpp	110.6 ± 1.5	0.056 ± 0.015	117.1 ± 2.0	0.047 ± 0.022
hexylmpp	110.2 ± 2.2	0.037 ± 0.036	115.9 ± 1.8	0.054 ± 0.061
butetpp	109.8 ± 1.9	0.059 ± 0.013	114.9 ± 2.4	0.081 ± 0.061
hexyletpp	110.3 ± 1.2	0.056 ± 0.011	116.3 ± 1.9	0.062 ± 0.061

**Table 2:** Main phase transition thermodynamic parameters (*T*_m_, Δ*T*_1/2_, and Δ*H*) of DMPC LUVs with 5 mM of chelator added ^a^.

	*T*_m_/°C	Δ*T*_1/2_/°C	Δ*H*/kJ·mol^−1^
DMPC (no ligand added)	24.4	0.8	20.2
DMPC + mpp	24.4	0.8	20.2
DMPC + dmpp	24.4	0.8	20.3
DMPC + etmpp	24.4	0.8	22.1
DMPC + butmpp	24.2	0.8	22.5
DMPC + hexylmpp	22.5	0.8	23.7
DMPC + MRB13	24.4	0.8	20.2
DMPC + etpp	24.4	0.8	20.2
DMPC + detpp	24.4	0.8	19.8
DMPC + butetpp	24.1	0.8	22.2
DMPC + hexyletpp	21.5	0.8	21.5
DMPC + MRE13	24.4	0.8	20.5

^a^ The estimated uncertainty is ±0.2 °C for the transition temperature, ±0.2 °C for the half width at half height, and ±1.5 kJ·mol^−1^ for Δ*H*.

**Table 3:** Main phase transition thermodynamic parameters (*T*_m_, Δ*T*_1/2_, and Δ*H*) for increased chelators concentrations in DMPC LUV’s ^a^.

	*T*_m_/°C	Δ*T*_1/2_/°C	Δ*H*/kJ·mol^−1^
DMPC (no chelator added)	24.4	0.8	20.2
DMPC + butmpp 2 mM	24.4	0.8	22.2
DMPC + butmpp 5 mM	24.2	0.8	22.5
DMPC + butmpp 10 mM	24.1	0.8	22.7
DMPC + hexylmpp 0.5 mM	24.4	0.8	20.3
DMPC + hexylmpp 2 mM	24.0	0.8	21.4
DMPC + hexylmpp 5 mM	23.5	0.8	23.7
DMPC + hexylmpp 10 mM	22.4	0.8	23.8
DMPC + butetpp 1 mM	24.4	0.8	22.0
DMPC + butetpp 2 mM	24.4	0.8	22.1
DMPC + butetpp 3 mM	24.1	0.8	22.1
DMPC + butetpp 5 mM	24.1	0.8	22.2
DMPC + hexyletpp 0.2 mM	24.3	0.8	20.8
DMPC + hexyletpp 0.5 mM	24.0	0.8	20.7
DMPC + hexyletpp 1 mM	23.4	0.8	21.6
DMPC + hexyletpp 5 mM	21.5	0.8	21.5

^a^ The estimated uncertainty is ±0.2 °C for the transition temperature, ±0.2 °C for the half width at half height, and ±1.5 kJ·mol^−1^ for Δ*H*.

## Data Availability

The synthesis and characterization of MRE13 can be found at ESI.

## Supplementary Materials

The following supporting information can be downloaded at: https://www.mdpi.com/article/10.3390/molecules29245905/s1, Figure S1: Mass spectrometry spectrum of compound MRE13; Figure S2: FTIR spectrum of compound MRE13: (a) full scan; (b,c) insets on relevant regions of the spectrum; Figure S3: NMR data relative to compound MRE13: (a) 1H, (b) 13C, (c) COSY, (d) HSQC and (e) HMBC spectra.
