# Supplementary material for: Exploring the Interaction of 3-Hydroxy-4-pyridinone Chelators with Liposome Membrane Models: Insights from DSC and EPR Analysis
Source: Molecules. 2024 Dec 14;29(24):5905. doi: 10.3390/molecules29245905 (PMC11676919; doi:10.3390/molecules29245905)
Supplement: Supplementary file 1 [file molecules-29-05905-s001.zip › molecules-3314949-supplementary.pdf]

**ESI**

# Exploring the interaction of 3-hydroxy-4-pyridinone chelators with liposome membrane models: Insights from DSC and EPR Analysis

Luísa M.P.F. Amaral[1]*, Tânia Moniz[1,2] and Maria Rangel[2]

[1] REQUIMTE, LAQV, Departamento de Química e Bioquímica, Faculdade de Ciências, Universidade do Porto, R. do Campo Alegre, 4169-007, Porto, Portugal; Luísa Amaral (luisaamaral@fc.up.pt)

[2] REQUIMTE, LAQV, Instituto de Ciências Biomédicas de Abel Salazar, Universidade do Porto, Rua Jorge Viterbo Ferreira, 228, 4050-313 Porto, Portugal; Tânia Moniz (tmoniz@icbas.up.pt); Maria Rangel (mrangel@icbas.up.pt)

*Correspondence: Luísa Amaral (luisaamaral@fc.up.pt)

## Methods

### Physical measurements

High resolution mass spectrometry (MS) analysis was done on an Orbitrap Exploris 120 mass spectrometer (Thermo Fischer Scientific, Bremen, Germany) controlled by Orbitrap Exploris Tune Application 2.0.185.35 and Xcalibur 4.4.16.14. The capillary voltage of the electrospray ionization source (ESI) was set to 3.4 kV and 2 kV for positive and negative mode. The capillary temperature was 320ºC. The sheath gas and auxiliary gas flow rate were at 5 (arbitrary unit as provided by the software settings). The resolution of MS scan was 60 000. The m/z range were 100-1200 Da. The resolution of SIM MS scan was 60,000. MS data handling software (Xcalibur QualBrowser software, Thermo Fischer Scientific) was used to search the expected molecule by their m/z value.

Fourier-transform infrared spectroscopy (FTIR) analysis was performed using Frontier FT-IR spectrophotometer (PerkinElmer, Beaconsfield, UK) equipped with an attenuated total reflectance (ATR) accessory. Spectrum was collected from 4000 to 600 $cm^{-1}$, considering 32 scans and a resolution of 4 $cm^{-1}$.

NMR spectra were recorded on a Bruker Avance III 400 spectrometer, operating at 400.15 MHz for $^{1}H$ and 100.62 MHz for $^{13}C$ atoms, equipped with pulse gradient units, capable

of producing magnetic field pulsed gradients in the z-direction of 50.0 G/cm. Two-dimensional $^{1}H/^{1}H$ correlation spectra (COSY), gradient selected $^{1}H/^{13}C$ heteronuclear single quantum coherence (HSQC) and $^{1}H/^{13}C$ heteronuclear multiple bond coherence (HMBC) spectra were acquired using the standard Bruker software.

**Synthesis of MRE13**

Firstly, the protected form of the ligand was obtained as previously described [https://doi.org/10.1016/j.poly.2018.12.005; https://pubs.acs.org/doi/10.1021/jm00069a002]. Briefly, a mixture of amine (3-methoxypropylamine) (2,32 mL, 0.0228 mol) and protected pyrone (3-benzyloxy-2-ethyl-4-pyrone) (1.75 g, 0.0076 mol) dissolved in water/ethanol 6:4 (10 mL) was placed in a 30 mL reaction vial, which was then closed under argon atmosphere and placed in the cavity of a CEM microwave reactor. The reaction vial was irradiated to 90ºC for 4 hours, using 100 W maximum power. The reaction solvent was evaporated, and the crude oil resultant was dissolved in 50 mL of H2O and the pH adjusted to 1 with HCl 10%. The starting materials were removed by liquid/liquid extraction with diethyl ether. The organic layer was rejected. The pH of aqueous phase was adjusted to 9 with a solution of NaOH 5% and the product was then extracted to the organic layer with dichloromethane. The organic phase was concentrated to afford compound MRE13p as yellow brown oil. The oil was dissolved in methanol (10 mL) and the solution was placed in a 50 ml round-bottom flask with a magnetic stirrer to which triethylsilane (10x) was added. The air was removed with N2, a catalytic amount of 10% Pd/C (w/w) was added, and the mixture was stirred overnight at room temperature.

The reaction mixture was then filtered at reduced pressure using a Buckner funnel with layer of celite and washed with methanol to completely remove the heterogeneous catalyst. The filtered solution was then concentrated to achieve a light brown crystal line solid, MRE13. The compound was purified by sublimation under reduced pressure.

## Results

**1-(3′-Methoxypropyl)-2-methyl-3-hydroxy-4-1H-pyridinone (MRE13):** MS: calculated for $C_{11}H_{18}NO_3^+$: 212.1281 (monoisotopic molecular weight $M^+$), found: HRMS: 212.1280 FTIR: freq ($cm^{-1}$): 2982, 2926, 2878 ($_{\nu OH}$); 1620, ($_{\nu C=O}$); 1566, 1508 ($_{\nu C=C}$); 1352, 1216, 1222 ($_{\nu C\text{-}H\ aromatic}$), 1120, 1036 ($_{\nu C\text{-}O}$), 972, 900, 846, 786, 756, 682, 638 ($_{\nu C\text{-}H\ aliphatic}$). 400.15 MHz $^1$H NMR (DMSO-$d_6$, ppm): δ 1.22 (t, *J* 7.4 Hz, 3H, 2-$CH_2C\underline{H}_3$), 1.88 (quint, *J* 6.2, 7.0 Hz, 2H, H2′), 2.69 (quart, *J* 7.4 Hz, 2H, 2-$C\underline{H}_2CH_3$), 3.25 (s, 3H, O-$C\underline{H}_3$), 3.32 (t, *J* 5.8 Hz, 2H, H3′), 3.96 (t, *J* 7.2 Hz, 2H, H1′), 6. 12 (d, *J* 7.6 Hz, 1H, H5), 7.51 (d, *J* 7.6 Hz, 1H, H6). 100.62 MHz $^{13}$C NMR (DMSO-$d_6$, ppm): δ 14.2 (2-$CH_2\underline{C}H_3$), 20.1 (2-$\underline{C}H_2CH_3$), 32.5 (C2′), 51.3 (C1′), 59.7 (O-$\underline{C}H_3$), 69.8 (C3′), 112.3 (C5), 135.1 (C2), 139.2 (C6), 146.7 (C3), 170.6 (C4).

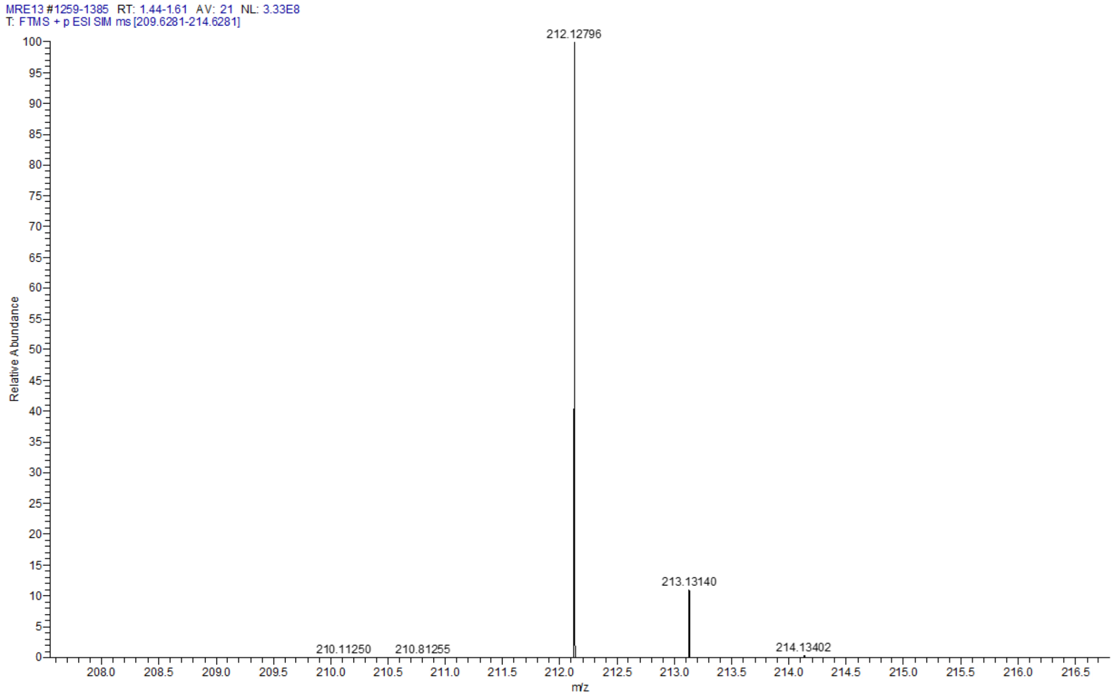


**Figure S1 –** Mass spectrometry spectrum of compound MRE13.

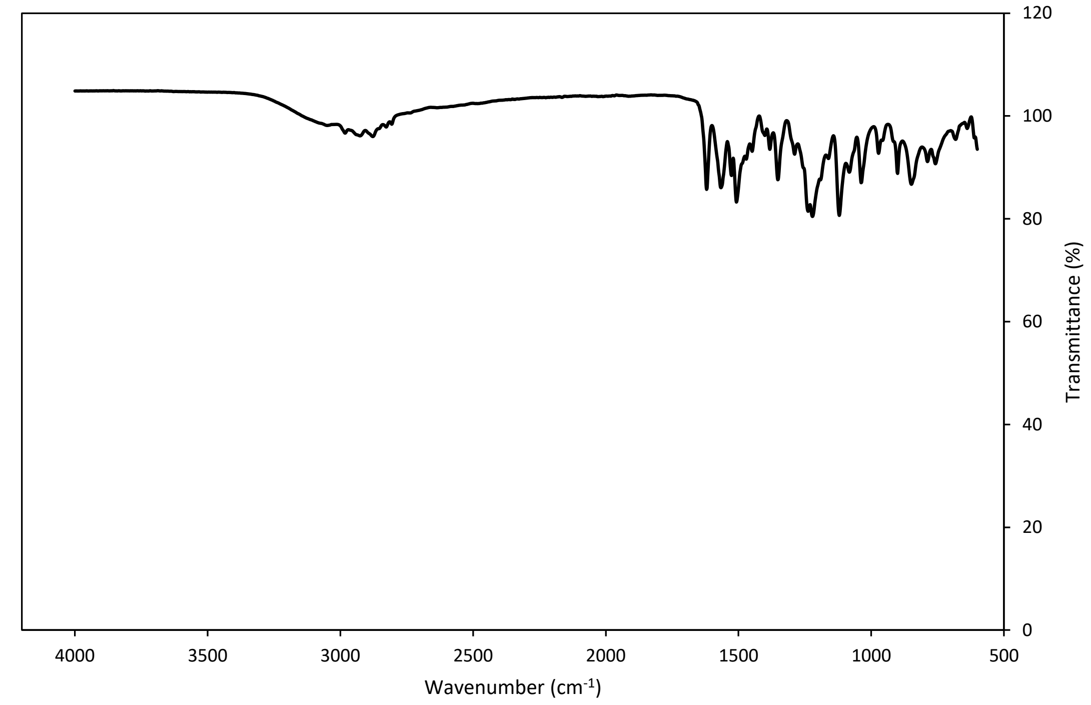

120
100
80
60
40
20
0
Transmittance (%)
4000
3500
3000
2500
2000
1500
1000
500
Wavenumber ($cm^{-1}$)


(a)

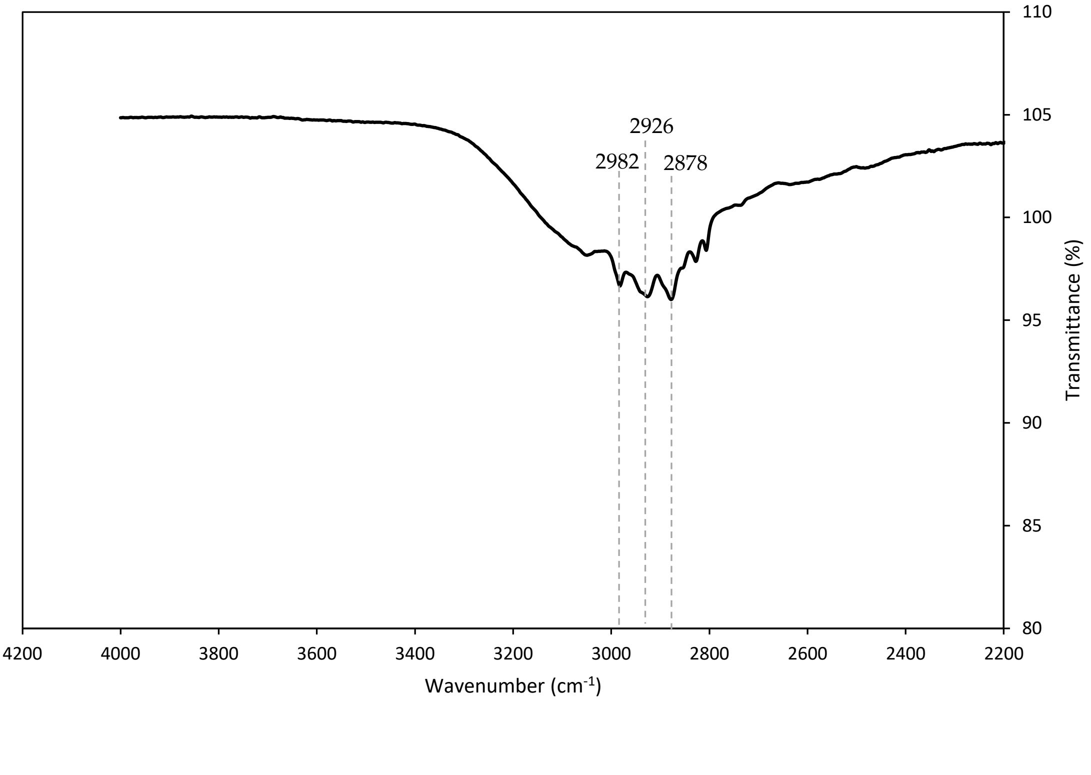

2926
2982
2878
110
105
100
95
90
85
80
Transmittance (%)
4200
4000
3800
3600
3400
3200
3000
2800
2600
2400
2200
Wavenumber ($cm^{-1}$)


(b)

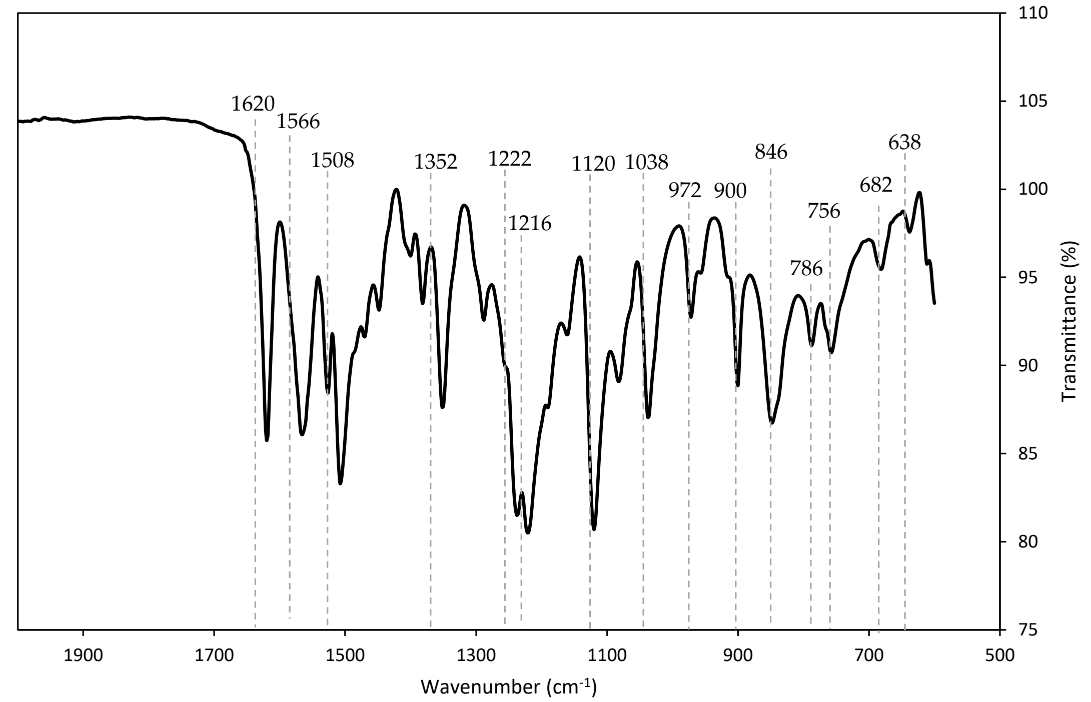


(c)

**Figure S2 –** FTIR spectrum of compound MRE13: a) full scan; b) and c) insets on relevant regions of the spectrum.

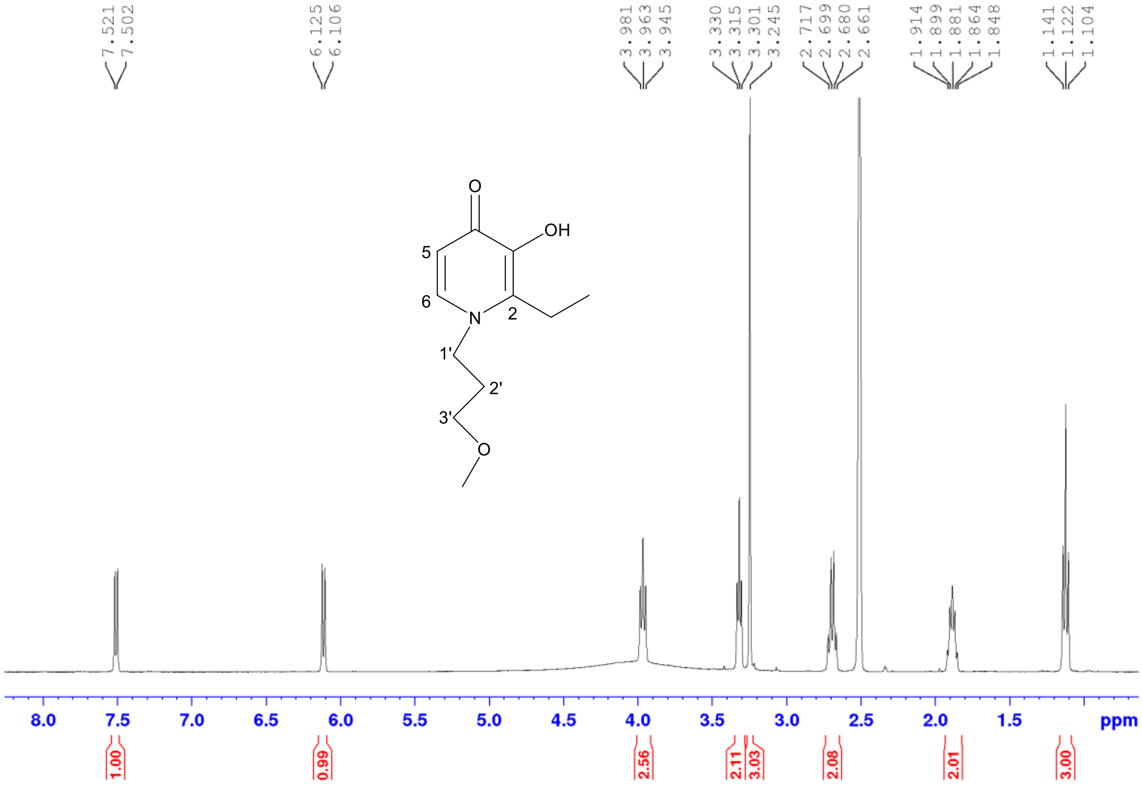
7.521
7.502
6.125
6.106
3.981
3.963
3.945
3.330
3.315
3.301
3.245
2.717
2.699
2.680
2.661
1.914
1.899
1.881
1.864
1.848
1.141
1.122
1.104
O
OH
5
6
N
2
1'
2'
3'
O
8.0
7.5
7.0
6.5
6.0
5.5
5.0
4.5
4.0
3.5
3.0
2.5
2.0
1.5
ppm
1.00
0.99
2.56
2.11
3.03
2.08
2.01
3.00

(a)

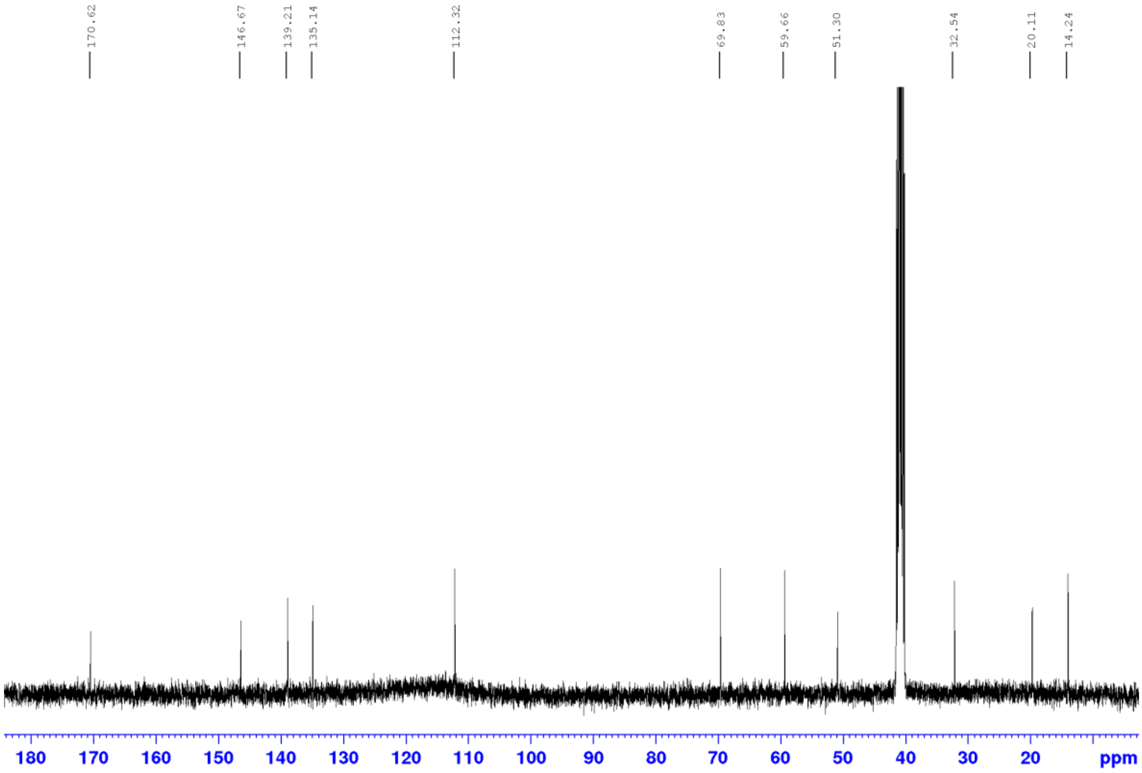
170.62
146.67
139.21
135.14
112.32
69.83
59.66
51.30
32.54
20.11
14.24
180
170
160
150
140
130
120
110
100
90
80
70
60
50
40
30
20
ppm

(b)

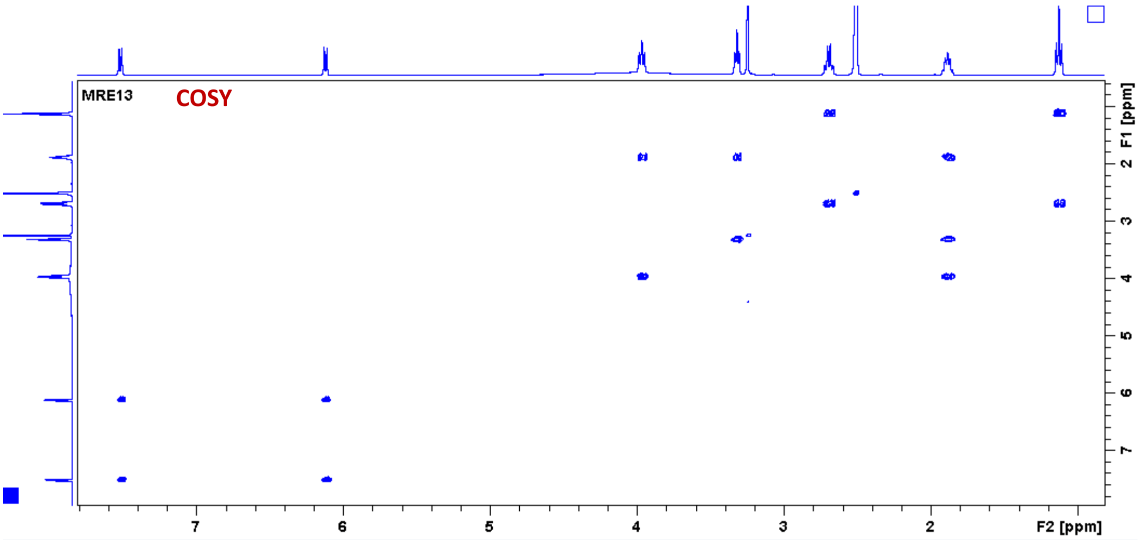


(c)

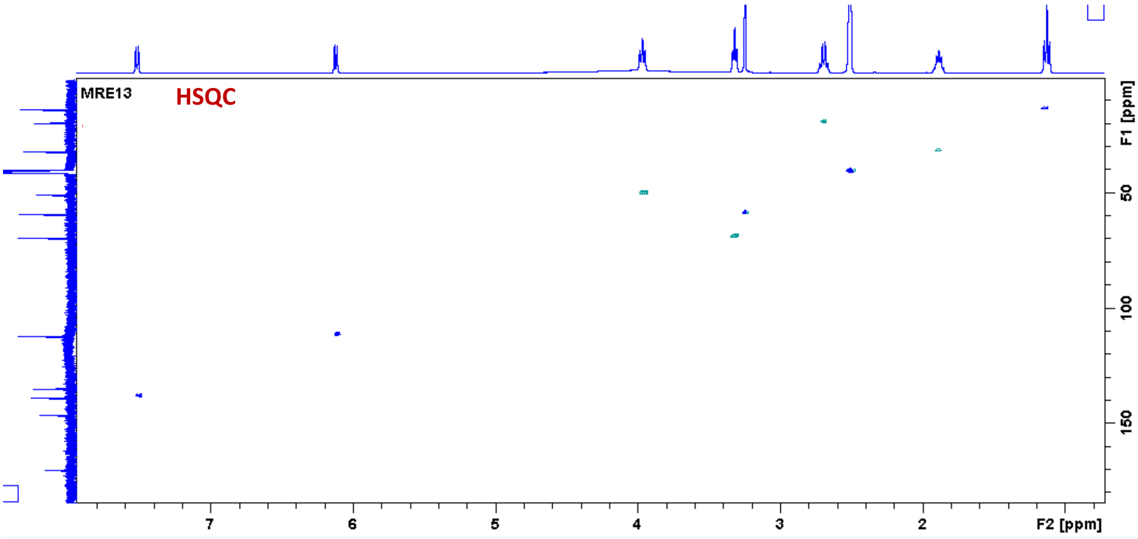


(d)

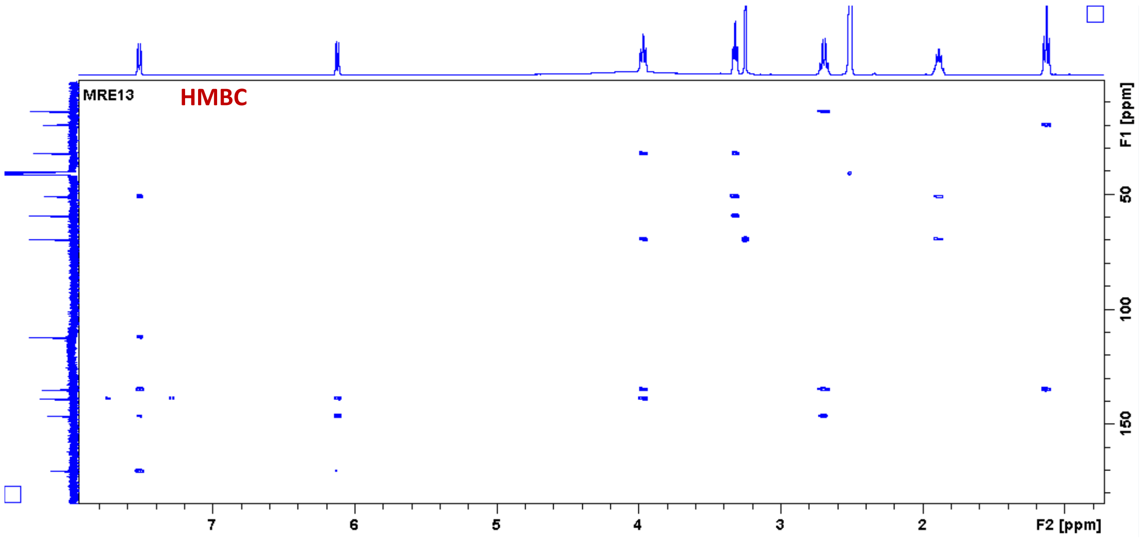


(e)

**Figure S3 –** NMR data relative to compound MRE13: a) $^{1}H$, b) $^{13}C$, c) COSY, d) HSQC and e) HMBC spectra.
